# Subtotal laparoscopic cholecystectomy versus conversion to open as a bailout procedure: a cohort study

**DOI:** 10.1007/s00464-024-10911-x

**Published:** 2024-07-09

**Authors:** Camilo Ramírez-Giraldo, Danny Conde Monroy, Andrés Isaza-Restrepo, Daniela Ayala, Juliana González-Tamayo, Ana Maria Vargas-Patiño, Luisa Trujillo-Guerrero, Isabella Van-Londoño, Susana Rojas-López

**Affiliations:** 1 Hospital Universitario Mayor - Méderi, Calle 24 #29 – 45, Bogotá, Colombia; 2 Universidad del Rosario, Bogotá, Colombia; 3 Grupo de Investigación Clínica, Escuela de Medicina y Ciencias de la Salud, Universidad del Rosario, Bogotá, Colombia

**Keywords:** Cholecystectomy, Laparoscopic, Difficult cholecystectomy, Complications

## Abstract

**Background:**

The aim of this study is to evaluate morbidity and mortality in patients taken to conversion to open procedure (CO) and subtotal laparoscopic cholecystectomy (SLC) as bailout procedures when performing difficult laparoscopic cholecystectomy.

**Method:**

This observational cohort study retrospectively analyzed patients taken to SLC or CO as bailout surgery during difficult laparoscopic cholecystectomy between 2014 and 2022. Univariable and multivariable logistic regression models were used to identify prognostic factors for morbimortality.

**Results:**

A total of 675 patients were included. Of the 675 patients (mean [SD] age 63.85 ± 16.00 years; 390 [57.7%] male) included in the analysis, 452 (67%) underwent CO and 223 (33%) underwent SLC. Overall, neither procedure had an increased risk of major complications (89 [19.69%] vs 35 [15.69%]* P*.207). However, CO had an increased risk of bile duct injury (18 [3.98] vs 1 [0.44]* P*.009), bleeding (mean [SD] 165.43 ± 368.57 vs 43.25 ± 123.42* P* < .001), intestinal injury (20 [4.42%] vs 0 [0.00]* P*.001), and wound infection (18 [3.98%] vs 2 [0.89%]* P*.026), while SLC had a higher risk of bile leak (15 [3.31] vs 16 [7.17]* P*.024). On the multivariable analysis, Charlson comorbidity index (odds ratio [OR], 1.20; CI95%, 1.01–1.42), use of anticoagulant agents (OR, 2.56; CI95%, 1.21–5.44), classification of severity of cholecystitis grade III (OR, 2.96; CI95%, 1.48–5.94), and emergency admission (OR, 6.07; CI95%, 1.33–27.74) were associated with presenting major complications.

**Conclusions:**

SLC was less associated with complications; however, there is scant evidence on its long-term outcomes. Further research is needed on SLC to establish if it is the safest in the long-term as a bailout procedure.

The current treatment of choice for benign biliary disease is laparoscopic cholecystectomy [1, 2]. Nonetheless, when faced with a difficult cholecystectomy during which the critical view of safety cannot be properly identified due to severe inflammation or fibrosis of the hepatocystic triangle, a bailout procedure is recommended [3] with the means to avoid complications such as bile duct injury [4].

Some of the currently defined bailout procedures for cholecystectomy available include conversion to open procedure (CO), subtotal laparoscopic cholecystectomy (SLC), cholecystostomy, a fundus-first approach, or, in worst-case scenarios, aborting the procedure if manipulation of the surgical area is too hazardous [3, 5]. Each of these approaches has its advantages and disadvantages; thus, the technique of choice during difficult cholecystectomy depends on the performing surgeon’s clinical criteria, experience, and expertise [5].

Cholecystostomy (drainage tube) is a minimally invasive, transient procedure employed in order to control the biliary focus of infection while the patient is taken to a second surgical procedure in an attempt to once again perform cholecystectomy as a means of avoiding symptomatic recurrence [6]. A fundus-first approach is more often associated with bile duct injury due to severe local inflammation where a clear dissecting plane between the cystic plate and porta hepatis cannot be appreciated; however, some studies refer to it as a safe alternative [5, 7]. On the other hand, aborting the procedure implies that a second deferred cholecystectomy attempt must be made, increasing morbidity. Due to all of the above, the currently preferred bailout procedures are CO and SLC; however, these procedures are not exempt from complications [8]. The current surgical tendency appears to favor performing SLC over CO as a bailout procedure, which may be associated with the trend that newer generation surgeons have more experience with laparoscopic surgery [9]. That being said, current literature where both of these techniques are compared is limited and as a result, it is necessary to delve into which procedure has better surgical outcomes as the safest bailout procedure when performing difficult cholecystectomy.

Owing to all the aforementioned, the aim of this study is to evaluate morbidity and mortality in patients taken to CO and SLC as bailout procedures when performing difficult laparoscopic cholecystectomy.

## Methods

### Study design

A retrospective observational cohort study was designed. We reviewed 16,225 cholecystectomies performed by a total of 35 surgeons with varying degrees of experience, which represent the total number of cholecystectomies performed at our institution between 2014 and 2022. We selected the preemptive conversion and subtotal laparoscopic cholecystectomy cases (bailout procedures), from which we obtained the information. Cholecystectomies that did not require bailout procedures, those that were reactive conversions, those that were scheduled as open procedures, those associated with another procedure, and 15 cases with incomplete information were excluded. All variables were collected in an anonymous database. This study was reviewed and approved by our institution’s ethics committee (number DVO005 2349-CV1737). The study was conducted under the principles of the Declaration of Helsinki [10]. We followed the STROBE guidelines to report this study [11].

### Patients

Patients with a preoperative diagnosis of gallbladder cancer, patients in which cholecystectomy was associated with another surgical procedure (such as gastrectomy or pancreatoduodenectomy), patients without postoperative follow-up of 30 days, patients in which reactive conversion was performed, and patients whose data registry did not include the predetermined variables of interest were excluded.

Preemptive conversion (elective conversion before a complication develops) differs from reactive conversion (emergency conversion due to an intraoperative complication) if the decision to convert is taken before or because of an intraoperative complication [12]. Both procedures are mutually exclusive. We decided to exclude reactive conversions because their results would not be comparable to SLC in terms of morbidity.

The indications for performing laparoscopic cholecystectomy were the following: all cases of benign biliary disease (biliary colic, pancreatitis, choledocholithiasis, cholecystitis, or a combination of them) where at least one diagnostic image study evidenced biliary disease. In cases of cholecystitis, this was diagnosed, classified, and managed following what is established in the Tokyo guidelines [13, 14]. Additionally, the American guidelines protocol for the risk of choledocholithiasis was followed; in low-risk cases, cholecystectomy was performed without the need for additional studies, in intermediate-risk cases a magnetic resonance cholangiopancreatography was performed, while in high-risk cases patients were taken to an endoscopic retrograde cholangiopancreatography (ERCP) previous performing cholecystectomy [15]. Cases of pancreatitis were managed according to both international and our institution’s guidelines, defining the timing of cholecystectomy when pancreatitis was clinically resolved [16, 17].

All patients had a scheduled outpatient control appointment where adequate clinical progress, healing of the surgical wounds, and the surgical specimen’s histopathology report were reviewed to ensure an adequate postoperative evolution or the need for further studies.

The following variables were analyzed: patients’ demographic characteristics, body mass index, ASA Physical Status Classification, previous diagnosis of either diabetes mellitus, arterial hypertension, chronic obstructive pulmonary disease, chronic kidney disease, cardiovascular disease and/or liver disease, current therapy with antiplatelet or anticoagulant agents, previous history of abdominal surgery, previous episodes of cholecystitis, preoperative blood work tests, surgical procedure indication, imaging findings on preoperative image studies, classification according to its severity (in cases of cholecystitis), the need for preoperative ERCP, history of cholecystostomy, type of admission, time interval between hospital admission and surgical procedure, type of incision (in cases of CO), type of subtotal cholecystectomy according to Purzner’s classification [18] (in cases of SLC), surgeon experience, need for drain placement, intraoperative findings according to Nassar’s modified score for difficult cholecystectomy [19], Clavien-Dindo’s score for complications associated with both the procedure and hospital admission [20], length of hospital stay, need for reintervention, and 30-day mortality.

### Surgical procedure

Laparoscopic cholecystectomy was performed in the American position using the standard 4-port technique [1 umbilical port, 1 subxiphoid port, and 2 ports in the right hypochondrium]. Dissection of the hepatocystic triangle was performed until the critical view of safety could be visualized, performing the dissection above Rouviere’s sulcus in a lateral to medial direction. In cases where the critical view of safety could not be properly reached, it was at the surgeon’s discretion to perform either SLC or CO. The need to place a drain in the surgical site was also decided according to the attending surgeon’s own criteria [21].

In cases of CO, the type of approach (by either a subcostal or midline incision) was also decided depending on the attending surgeon’s preference. A fundus-first approach was then performed until the gallbladder was completely liberated from the cystic plate, followed by proper identification of both the cystic duct and artery. In cases where the structures from the hepatocystic triangle could not be safely identified, subtotal open cholecystectomy or cholecystostomy was performed.

On the other hand, when SLC was performed, the gallbladder wall portion free from the liver was opened with either a fundus-first downward or body-upward approach using electrocautery, hoping to dissect as much as possible of this free gallbladder wall while staying above the safety line between Rouviere’s sulcus and the umbilical fissure [5]. If the cystic artery was identified, it was clipped during the division of the gallbladder wall; although on multiple occasions, it could not be properly identified due to the severe inflammatory process, during which it was probably in a thrombotic state. All gallstones would then be extracted from the remaining gallbladder portion. If the cystic duct’s orifice was visible, it was sutured; however, due to severe inflammation this was not possible in the majority of cases. Consequently, the gallbladder stump was also electrocauterized. In cases where the surgeon’s experience and personal decision allowed, the remaining gallbladder stump was closed with sutures. Likewise, removing the gallbladder wall in contact with the liver or leaving it in situ was also according to the surgeon’s personal decision intraoperatively [22, 23].

### Statistical analysis

A description of demographical, clinical, paraclinical, intraoperative, and surgical outcome variables was performed. Categorical variables were described as proportions, whereas continuous variables were described as means and standard deviations (SD). The incidence for both SLC and CO was calculated for each year between 2014 and 2022. A univariate analysis was made, using the Chi-squared test in the case of categorical variables and the 2-tailed *t* test with continuous variables to evaluate the differences between them depending on the type of bailout procedure performed and type of subtotal cholecystectomy. A logistical regression model was built in order to identify factors associated with major complications (defined as a Clavien-Dindo score ≥ 3). All variables that were clinically relevant were included in the binary logistic regression model. Both crude odds ratios (OR) and the adjusted OR to their respective 95% confidence intervals (CI95%) were calculated to determine the magnitude of association between risk factors identified in the multivariate analysis for morbimortality. Data analysis was performed from July to September 2023. The analyses were conducted using SPSS 29 statistical software and the RStudio.

## Results

**Fig. 1:**
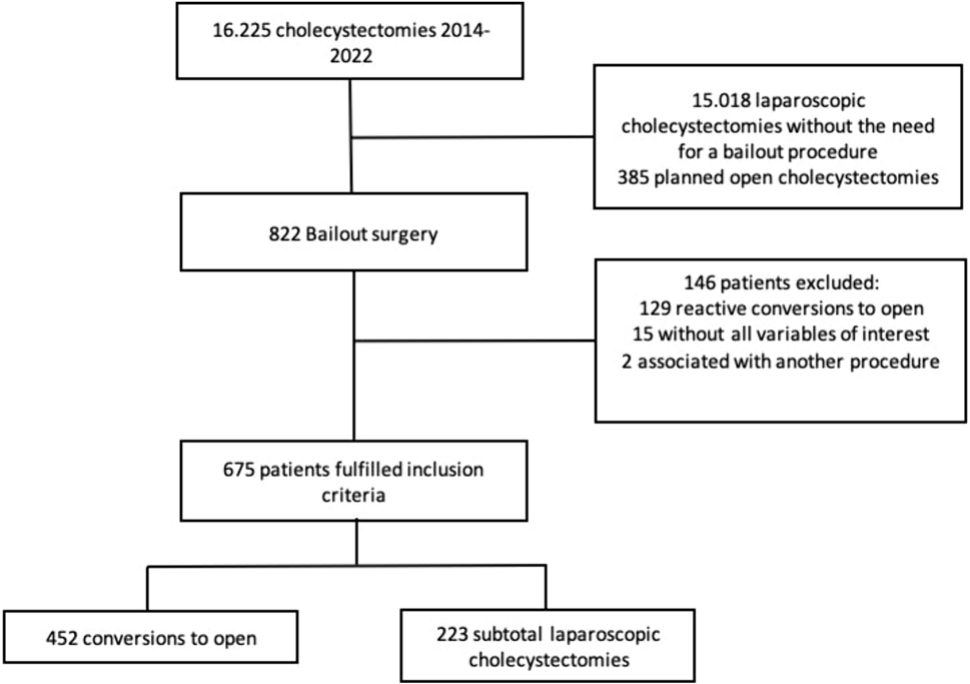
Study selection process flowchart

A total of 675 patients taken to cholecystectomy which was considered difficult and thus required a bailout procedure were included in this study, out of which 452 of them were CO and 223 were SLC. The study selection process can be seen in the following flowchart (Fig. 1).

**Fig. 2:**
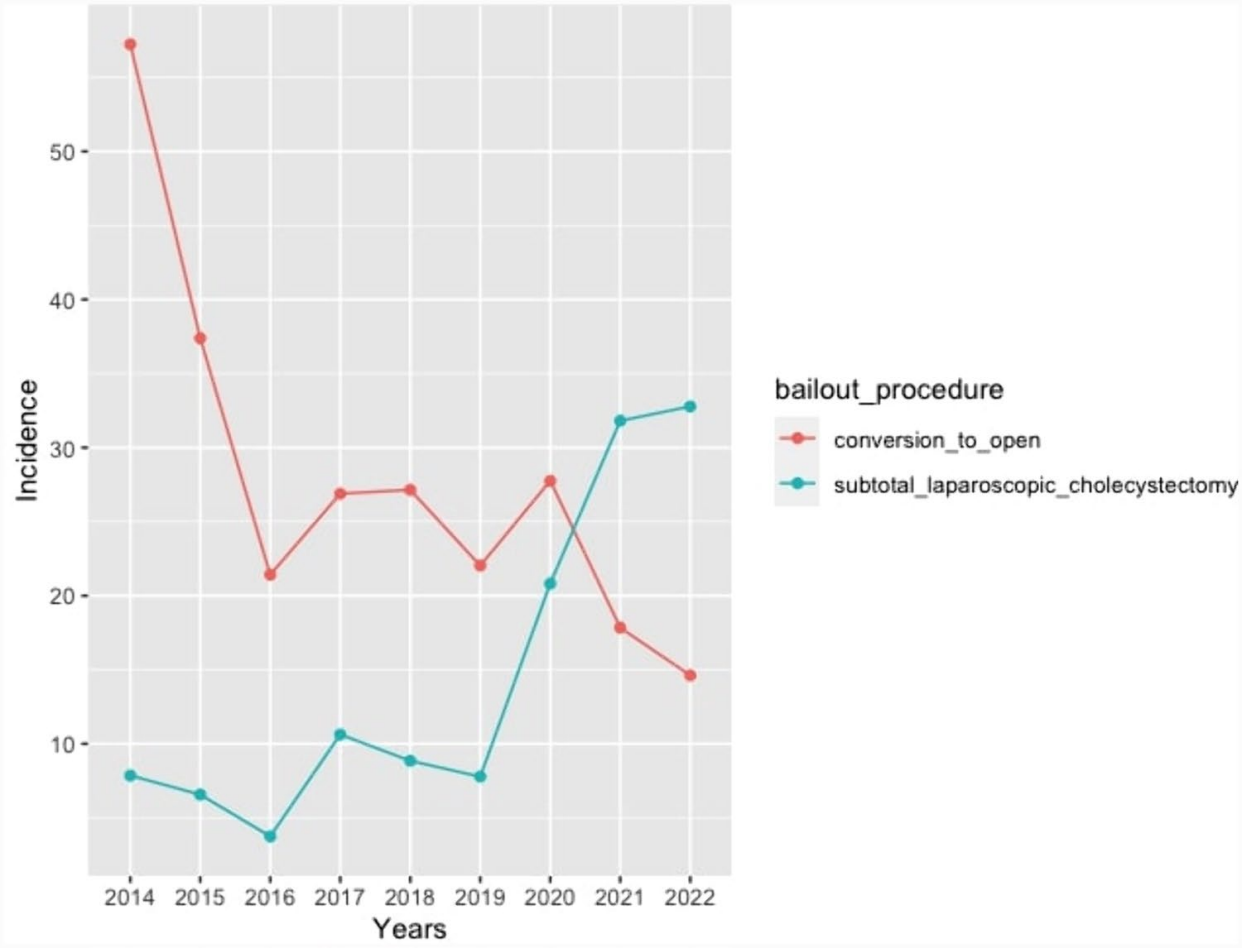
Incidence of bailout procedures for every 1000 cholecystectomies per year

The incidence for bailout surgery was 42.58 for every 1000 laparoscopic cholecystectomies performed. When observing each year between 2014 and 2022, CO has a higher incidence as the preferred bailout procedure in 2014 while SLC is more frequent in 2022. This change of incidence happened linearly, finding a progressive reduction of CO and an increase in SLC across time (Fig. 2).

The age mean was of 63.85 ± 16.00 years and patients’ sex was predominantly male (57.77%). The most frequent diagnosis for performing either type of bailout procedure was cholecystitis (68.88%), mainly grade II cholecystitis according to the Tokyo guidelines (41.92%), followed by grade I (14.81%) [13, 14]. On patients where CO was performed, 169 (37.38%) of them ended up with an open subtotal cholecystectomy and 3 (0.66%) of them with cholecystostomy. Most cholecystectomies were difficult according to the intraoperative findings described in Nassar’s modified scale (97.99%) [19]. Other demographic, clinical, and surgical characteristics are described in Tables 1 and  2.

**Table 1:** Demographic and clinical characteristics according to bailout procedure performed

	*N* (%)	Conversion to open procedure (*n* = 452)	Subtotal laparoscopic cholecystectomy (*n* = 223)	*p* value
Age (mean)(SD)(years)	63.85﻿ ± 16.00	64.31 ± 15.40	62.91 ± 17.15	0.284*
Sex				
Female Male	285 (42.22) 390 (57.77)	191 (42.25) 261 (57.74)	94 (42.15) 129 (57.84)	0.979
Body mass index (mean)(SD)(kg/m^2^)	26.94 ± 4.67	26.99 ± 4.86	26.85 ± 4.29	0.740*
ASA classification				
1 2 3 4–5	79 (15.11) 270 (51.63) 162 (30.98) 12 (2.29)	59 (13.05) 187 (41.37) 100 (53.47) 11 (5.88)	20 (12.50) 83 (51.88) 56 (35.00) 1 (0.62)	0.169
Co-morbidity				
Diabetes mellitus Arterial hypertension Chronic obstructive pulmonary disease Chronic kidney disease Cardiovascular disease Liver disease	148 (22.02) 333 (49.55) 60 (8.93) 30 (4.46) 116 (17.29) 10 (1.49)	100 (22.12) 225 (44.44) 39 (8.62) 19 (4.20) 76 (16.81) 5 (1.10)	48 (21.52) 108 (48.43) 21 (9.42) 11 (4.93) 40 (17.94) 5 (2.24)	0.826 0.682 0.754 0.679 0.754 0.257
Charlson comorbidity index				
(Mean)(SD)(points)	2.65 ± 1.90	2.71 ± 1.85	2.54 ± 2.01	0.281
Anticoagulant agents	38 (5.65)	25 (5.56)	13 (5.83)	0.890
Antiplatelet agents	99 (14.73)	67 (14.92)	32 (14.35)	0.844
Previous surgical history				
Upper abdomen	81 (12.04)	63 (14.00)	18 (8.07)	**0.026**
Pre-operative laboratories (mean)(SD)				
Leukocytes (× 10^3^) Hemoglobin (mg/dL) Bilirubin (mg/dL) Alkaline phosphatase (mg/dL) Aspartate aminotransferase (mg/dL) Alanine aminotransferase (mg/dL)	12.67 ± 5.11 14.62 ± 2.03 2.86 ± 15.87 198.59 ± 264.58 95.90 ± 153.38 111.13 ± 166.00	12.95 ± 5.18 14.69 ± 2.03 3.26 ± 19.45 182.63 ± 161.27 91.52 ± 159.31 102.90 ± 162.10	12.11 ± 4.92 14.49 ± 2.04 2.10 ± 2.36 230.69 ± 396.81 104.20 ± 141.46 126.73 ± 172.47	0.054* 0.258* 0.387* 0.035* 0.334* 0.092*
Imaging findings				
Bile duct diameter (mean)(SD)(mm) Gallbladder wall thickness (mean)(SD)(mm) Signs of cholecystitis Abscess or pericholecystic fluid Perforation Scleroatrophic gallbladder Impacted stone	5.79 ± 2.32 3.61 ± 1.86 443 (66.42) 133 (19.97) 30 (4.50) 14 (2.10) 61 (9.15)	5.77 ± 2.23 3.57 ± 1.81 287 (63.70) 87 (19.57) 17 (3.70) 11 (2.43) 28 (6.19)	5.81 ± 2.51 3.67 ± 1.97 156 (70.27) 46 (20.72) 13 (5.86) 3 (1.79) 33 (14.86)	0.831* 0.507* 0.137 0.732 0.234 0.341 ** < 0.001**

Bold values indicate statistically significant p values (*p* < 0.05)

*p* values were obtained using the Chi-squared test

*p values were obtained using the 2-tailed *t*

**Table 2:** Pathology and surgical characteristics according to bailout procedure performed

	*N* (%)	Conversion to open procedure (*n* = 452)	Subtotal laparoscopic cholecystectomy (*n* = 223)	*p* value
Surgical procedure indication				
Biliary colic Pancreatitis Choledocholithiasis Acute cholecystitis	134 (19.85) 62 (9.18) 116 (17.18) 465 (68.88)	101 (23.34) 46 (10.17) 68 (15.04) 302 (66.8)	33 (14.79) 17 (7.62) 48 (21.52) 163 (73.09)	**0.019** 0312 **0.039** 0.105
Pre-operative ERCP				
No Yes	538 (80.06) 134 (19.94)	369 (81.63) 80 (17.69)	169 (75.78) 54 (24.22)	0.051
Type of admission				
Elective Delayed Emergency	75 (11.16) 581 (86.46) 16 (2.38)	55 (12.16) 382 (84.51) 12 (2.65)	20 (8.97) 199 (89.24) 4 (1.79)	0.330
History of cholecystostomy				
No Yes	659 (97.63) 16 (2.37)	446 (98.67) 6 (1.32)	213 (95.52) 10 (4.48)	**0.011**
Time from admission to surgical procedure (mean)(SD)(days)	4.73 ± 5.48	4.49 ± 5.11	5.21 ± 6.16	0.107*
Subtotal cholecystectomy				
No Yes	280 (41.48) 395 (58.52)	283 (62.61) 172 (37.38)	0 (0.00) 223 (100.00)	** < 0.001**
Intraoperative cholecystostomy				
No Yes	672 (99.55) 3 (0.44)	449 (99.33) 3 (0.66)	223 (100.00) 0 (0.00)	0.223
Drain placement				
No Yes	282 (41.78) 393 (58.22)	208 (46.01) 244 (53.98)	74 (33.18) 149 (66.82)	**0.001**

Bold values indicate statistically significant p values (*p* < 0.05)

*p* values were obtained using the Chi-squared test

*p values were obtained using the 2-tailed *t* test

**Fig. 3:**
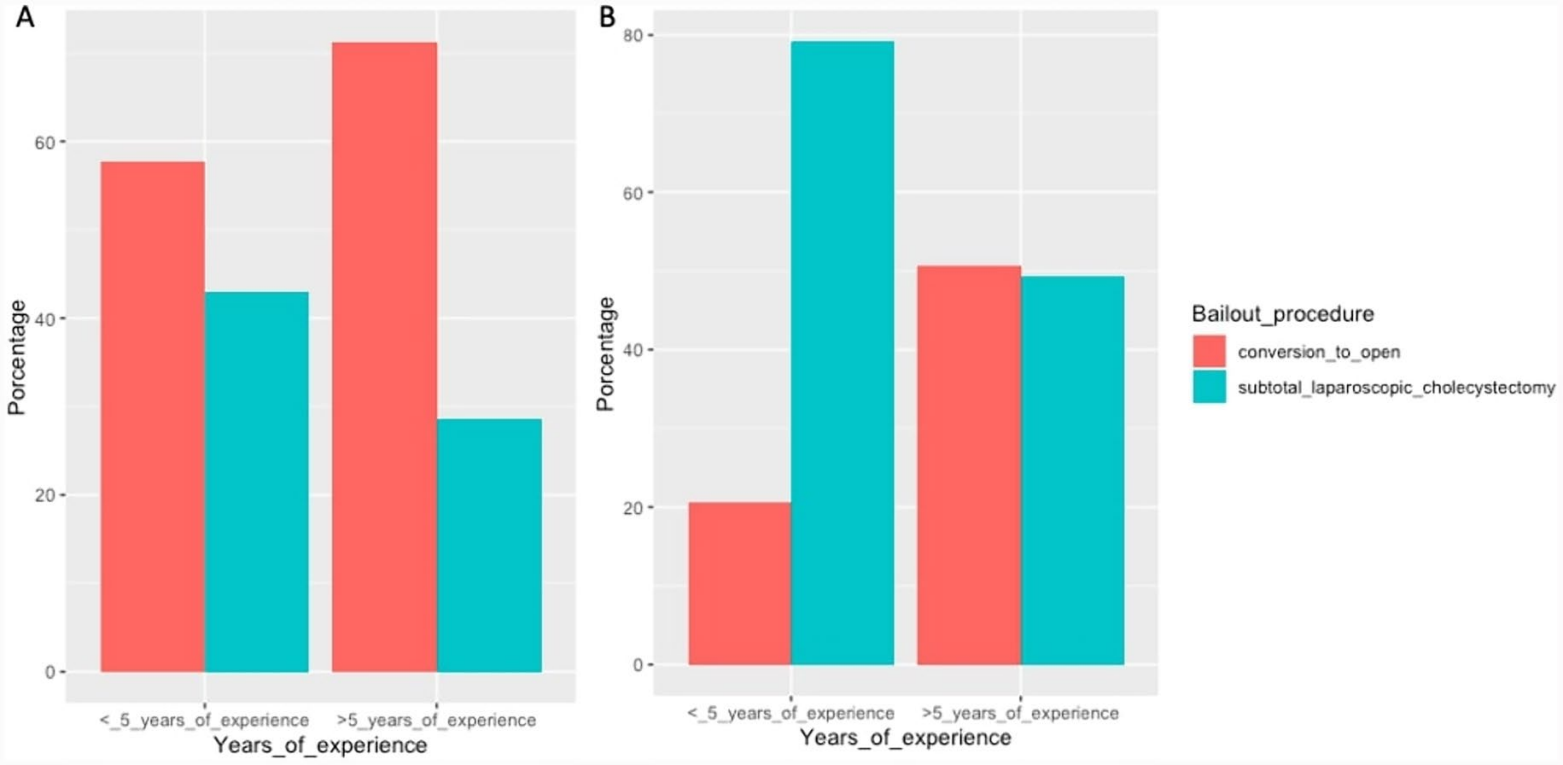
Bailout procedure according to the number of years of experience. **A** Period between 2014 and 2022. **B** Period between 2020 and 2022

When we evaluate the proportion of CO versus SLC based on whether the surgeons had more or equal to 5 years of experience, we observe that surgeons with more or equal to 5 years had a higher proportion of CO (72.56%) compared to surgeons with less than 5 years (26.79%), with a statistically significant difference (< 0.001). This disparity is steeper and can be observed more clearly between 2020 and 2022 (Fig. 3).

In terms of surgical outcomes, a higher, statistically significant proportion of bile duct injury, intraoperative bleeding, intestinal injury, and wound infection was evidenced in patients taken to CO. Nonetheless, there were no statistically significant differences when evaluating the rate of complications according to the Clavien-Dindo classification, including major complications, reintervention, and mortality (Table 3).

The analysis of surgical outcomes of subtotal cholecystectomies, categorized as reconstituting or "closed-tract" versus fenestrating or "open-tract," is presented in Table 4.

**Table 3:** Surgical outcomes according to bailout procedure performed

	*N* (%)	Conversion to open procedure (*n* = 452)	Subtotal laparoscopic cholecystectomy (*n* = 223)	*p* value
Hospital stay (mean)(SD) (days)	9.91 ± 9.44	9.96 ± 9.57	9.79 ± 9.19	0.827*
ICU stay (mean)(SD) (days)	0.77 ± 2.59	0.91 ± 2.90	0.48 ± 1.78	0.044*
Bile leak				
No Yes	609 (90.22) 66 (9.77)	415 (91.81) 37 (8.18)	194 (86.99) 29 (13.00)	**0.047**
Post-operative ERCP				
Choledocholithiasis Bile leak	16 (2.37) 31 (4.59)	10 (2.12) 15 (3.31)	6 (2.69) 16 (7.17)	0.701 **0.024**
Complications				
Bile duct injury	19 (2.81)	18 (3.98)	1 (0.44)	**0.009**
Bleeding (mean)(SD)(mL)	124.89 ± 314.72	165.43 ± 368.57	43.25 ± 123.42	< 0.001*
Bilioperitoneum	8 (1.18)	4 (0.88)	4 (1.79)	0.305
Intestinal injury	20 (2.96)	20 (4.42)	0 (0.00)	**0.001**
Wound infection	20 (2.96)	18 (3.98)	2 (0.89)	**0.026**
Fluid collection	20 (2.96)	16 (3.53)	4 (1.79)	0.208
Perioperative acute myocardial infarction	6 (0.88)	4 (0.88)	2 (0.89)	0.988
Perioperative venous thromboembolism	14 (2.07)	9 (1.99)	5 (2.24)	0.830
Health care-associated pneumonia	7 (1.03)	4 (0.88)	3 (1.34)	0.579
Health care-associated urinary tract infection	1 (0.14)	0 (0.00)	1 (0.44)	0.154
Pleural effusion	18 (2.66)	10 (2.21)	8 (3.58)	0.299
Reintervention				
No Yes	626 (92.74) 49 (7.25)	416 (92.03) 36 (7.96)	210 (94.17) 13 (5.82)	0.315
Clavien-Dindo				
I II IIIA IIIB IV V	43 (6.37) 47 (6.96) 23 (3.40) 54 (8.00) 28 (4.14) 19 (2.81)	28 (6.19) 39 (8.62) 14 (3.09) 40 (8.84) 21 (4.64) 14 (3.09)	15 (6.72) 8 (3.58) 9 (4.03) 14 (6.27) 7 (3.13) 5 (2.24)	0.065
Major complication				
No Yes	551 (81.62) 124 (18.37)	363 (80.30) 89 (19.69)	188 (84.30) 35 (15.69)	0.207
30-day mortality				
No Yes	656 (97.18) 19 (2.81)	438 (96.90) 14 (3.09)	218 (97.75) 5 (2.24)	0.527

Bold values indicate statistically significant p values (*p* < 0.05)

*p *values were obtained using the Chi-squared test

*p values were obtained using the 2-tailed *t* test

**Table 4:** Surgical outcomes according to reconstituting or “closed-tract” versus fenestrating or “open-tract”

	*N* (%)	Reconstituting or “closed-tract” (*n* = 335)	Fenestrating or “open-tract” (n = 60)	*p* value
Approach				
Laparoscopic Open	223 (56.46) 172 (43.54)	180 (53.73) 155 (46.27)	43 (71.66) 17 (28.33)	**0.010**
Hospital stay (mean)(SD) (days)	10.44 ± 9.03	10.25 ± 8.99	11.50 ± 9.24	0.442*
ICU stay (mean)(SD) (days)	0.74 ± 2.58	0.69 ± 2.57	1.00 ± 2.68	0.141*
Bile leak				
No Yes	342 (86.58) 53 (13.42)	294 (87.76) 41 (12.24)	48 (80.00) 12 (20.00)	0.104
Post-operative ERCP				
Choledocholithiasis Bile leak	11 (2.78) 28 (7.08)	9 (2.68) 19 (5.67)	2 (3.33) 9 (15.00)	0.779 **0.010**
Reintervention				
No Yes	368 (93.16) 27 (6.84)	315 (94.02) 20 (5.97)	53 (88.33) 7 (11.66)	0.107
Major complication				
No Yes	325 (82.28) 70 (17.72)	281 (83.88) 54 (16.12)	44 (73.33) 16 (26.66)	**0.049**
30-day mortality				
No Yes	384 (97.22) 11 (2.78)	326 (97.31) 9 (2.69)	58 (96.66) 2 (3.33)	0.779

Bold values indicate statistically significant p values (*p* < 0.05)

*p* values were obtained using the Chi-squared test

*p values were obtained using the 2-tailed *t* test

Most subtotal cholecystectomies were performed laparoscopically (56.46%), and the majority were reconstructive or "closed-tract"; of these reconstructive or "closed-tract" procedures, the majority were performed using an open approach. Major complications were more frequent in the open approach group (20.34% versus 15.69%), with no statistically significant differences.

We performed a binary logistic regression model to assess the factors associated with major complications (Clavien-Dindo score ≥ 3).

The model showed that Charlson comorbidity index, use of anticoagulant agents, severity of cholecystitis, and emergency admission were associated with major complications (Clavien-Dindo ≥ 3) (Table 5).

**Table 5:** Binary regression model for major complications (Clavien-Dindo ≥ 3)

	Crude OR (CI95%)	Multivariable adjusted OR (CI95%) *	Multivariable adjusted OR (CI95%) **
Age	1.02 (1.01–1.03)	1.00 (0.98–1.02)	
Sex			
Female Male	1.00 (referent) 1.10 (0.74–1.63)	1.00 (referent) 0.96 (0.63–1.47)	
Type of cholecystectomy			
Total Subtotal reconstituting Subtotal fenestrating	1.00 (referent) 0.80 (0.53–1.21) 1.52 (0.79–2.90)	1.00 (referent) 0.73 (0.43–1.23) 1.24 (0.55–2.76)	1.00 (referent) 0.74 (0.44–1.25) 1.34 (0.60–2.95)
Bailout procedure approach			
Laparoscopic Open	1.00 (referent) 1.31 (0.85–2.02)	1.00 (referent) 1.32 (0.75–2.31)	1.00 (referent) 1.35 (0.77–2.36)
Charlson comorbidity			
Index	**1.26 (1.14–1.40)**	**1.20 (1.01–1.42)**	**1.21 (1.08–1.35)**
Classification of severity of cholecystitis			
Not apply I II III	1.00 (referent) 1.93 (0.98–3.79) **2.26 (1.32–3.86)** **3.80 (1.98–7.29)**	1.00 (referent) 1.88 (0.92–3.81) 1.75 (0.99–3.11) **2.96 (1.48–5.94)**	1.00 (referent) **2.11 (1.05–4.24)** **2.09 (1.20–3.65)** **3.62 (1.84–7.15)**
Anticoagulant agents			
No Yes	1.00 (referent) **3.54 (1.80–6.96)**	1.00 (referent) **2.56 (1.21–5.44)**	1.00 (referent) **2.64 (1.27–5.46)**
Type of admission			
Elective Delayed Emergency	1.00 (referent) **4.28 (1.53–11.98)** **10.65 (2.55–44.40)**	1.00 (referent) 2.71 (0.92–7.94) **6.07 (1.33–27.74)**	

Bold values indicate statistically significant p values (*p* < 0.05)

*Multivariable model includes age, sex, type of cholecystectomy, approach, Charlson comorbidity index, classification of severity of cholecystitis, anticoagulant agents, and type of admission

**Multivariable model includes type of cholecystectomy, approach, Charlson comorbidity index, classification of severity of cholecystitis, and anticoagulant agents

## Discussion

The incidence of bailout surgery in cholecystectomy was 42.58 per every 1000 laparoscopic cholecystectomies performed in the population analyzed during the study’s time period. This rate of bailout surgery coincides with the ones already described in the current literature [12, 24]. Patients on which bailout cholecystectomy surgery was performed had a higher age average (63.85 ± 16.00), were predominantly male and overweight (BMI of 26.94 ± 4.67), and most had a severity diagnosis of grade II or III cholecystitis; all of these variables have been formerly reported as risk factors associated with difficult cholecystectomy [25–27].

Conversion rates progressively diminished over time, and on the contrary, the rates for SLC increased. This phenomenon can be explained considering that modernly trained younger surgeons have a lower degree of expertise in open cholecystectomy due to lower exposure to this approach in relation to the exponential popularity of laparoscopic surgery during the last century. Nonetheless, surgeons with more years of experience were most likely trained in open cholecystectomy as the preferred surgical approach for benign biliary disease and would thus favor this technique as their bailout procedure of choice over SLC. This can be evidenced in the rate of CO according to surgeon experience, where surgeons with < 5 years of experience had rates of 26.79% versus rates of 72.56% in surgeons with over 5 years of experience [9, 28]. Moreover, some of the most recent expert recommendations prefer SLC over CO as a bailout procedure, which may bias younger surgeons [8]. Another factor that may be related to higher CO rates is previous history of upper abdomen surgery, evidenced as statistically significant in our sample. A probable explanation for this may be that visceral adherence associated with previous surgical history may prevent the attending surgeon from performing SLC in spite of it being their procedure of choice [29].

Another important factor to note was that on patients taken to CO, 37.38% of procedures ended up as open subtotal cholecystectomies while 0.66% ended up as cholecystostomies. Some authors consider that CO is not a bailout procedure per se when faced with difficult cholecystectomy but rather what results from the procedure after conversion, which could trifurcate into either total open cholecystectomy, subtotal open cholecystectomy, or cholecystostomy [8]. Considering the aforementioned high rates of subtotal open cholecystectomy in procedures that were CO, one can conclude that conversion could be avoided and that subtotal cholecystectomy could be better performed using a laparoscopic approach. When performing subtotal cholecystectomy with a converted open approach, the benefits usually associated with laparoscopic surgery would be lost [30–32] (less postoperative pain and lesser rates of intra- and postoperative complications such as bleeding, bile duct injury, surgical wound infection, dehiscence, among others).

In a recently published study where SLC is compared with CO, CO is suggested as the preferred surgical alternative due to similar rates of complications, time of hospital stay, and readmissions. However, this approach presented higher rates of reinterventions compared with SLC. Additionally, when analyzing the results of this study we can observe that a CO does not necessarily guarantee a complete cholecystectomy [33].

The results of this study evidenced that there was not a statistically significant difference between patients taken to either CO or SLC on rates of major complications, reinterventions, time of hospital stay, and mortality. Furthermore, in patients taken to an open procedure, there was a statistically significant higher proportion of bile duct injury, intraoperative bleeding, and intestinal injury. As such it can be observed that a conversion will not necessarily guarantee the absence of bile duct injury, which is one of the arguments in the reported literature that favors SLC over CO.

In another study where the surgical outcomes for CO and SLC were compared, CO was associated with higher rates of bile duct injury, bleeding, postoperative ileus, ICU admission, and longer hospital stay. However, this cohort of patients included reactive conversions (emergent conversion due to an intraoperative complication), as such this sample may be biased and is probably the reason for the more unfavorable surgical outcomes associated with CO in this study [34].

When analyzing subtotal cholecystectomies, the fenestrating or "open-tract" technique was the most frequent choice when the approach was laparoscopic. It is important to highlight that this approach had a higher proportion of bile leakage and major complications compared to reconstituting or "closed-tract" cholecystectomy, a finding that is consistent with what has been published in the literature [35–38]. We can hypothesize that the choice of this technique is due to the technical difficulty involved in closing the stump via minimally invasive methods.

This study found that risk factors for major complications during bailout surgery were Charlson comorbidity index, use of anticoagulant agents, classification of severity of cholecystitis, and the type of admission. These variables have already been reported as risk factors associated with the difficulty of cholecystectomy and higher perioperative morbimortality [39, 40]. On the other hand, the type of bailout procedure (CO versus SLC) and the type of subtotal cholecystectomy (reconstituting or "closed-tract" versus fenestrating or "open-tract") did not emerge as factors associated with a higher proportion of major complications in the multivariate analysis.

Subtotal cholecystectomy has gained popularity as the preferred bailout surgery of choice when faced with difficult cholecystectomy [41] although this study did not evidence any superiority between SLC and CO for the appearance of major complications and mortality. Nevertheless, there was a significantly higher proportion of bile duct injury, intraoperative bleeding, and intestinal injury associated with conversion. Furthermore, CO does not guarantee to perform a total cholecystectomy, and we can then hypothesize this may add up to a subtotal cholecystectomy’s morbidity if the procedure is converted. It is also important to consider that the evidence on long-term outcomes for SLC is limited, as current literature is lacking on the risk of recurrence and malignancy of the remaining gallbladder stump [42]. As such it is necessary to broaden current research on which bailout surgical procedures for difficult cholecystectomy yield better outcomes and as a result be able to establish a definite recommendation on the subject.

This study has several distinct strengths. To our knowledge of current literature, it contains the most extensive series that compares surgical outcomes for SLC and CO as bailout procedures when faced with difficult cholecystectomy. Moreover, it excludes reactive conversions. The most relevant limitation of this study is its retrospective design and the surgeon's discretionary decision to perform the bailout procedure, which makes it prone to selection bias. Another significant limitation is that follow-up was only conducted at 30 days, so long-term outcomes cannot be assessed, and these are important when determining the best procedure.

## Conclusions

SLC seems to be a good alternative when faced with difficult cholecystectomy; however, it is necessary to take into account the limitations of current scientific knowledge on its long-term outcomes such as recurrence and malignancy. Conversely, in terms of CO, this procedure is still an alternative therapeutic approach to consider; however, it has seen a decline considering that more recently trained surgeons have lower exposure to the technique due to the current predominantly laparoscopic approach to cholecystectomy. Additionally, performing an open conversion does not necessarily mean the procedure will end as a total cholecystectomy, and a significant proportion of patients will end up having a subtotal cholecystectomy either way with the added morbidity of an open approach in comparison with laparoscopic surgery.

## Data Availability

Data are available in 10.34848/LP51IE.
